# Review of Neil Selwyn, Felicitas Macgilchrist, and Ben Williamson (2020). Digital Education after COVID-19. *TECHLASH, 1*

**DOI:** 10.1007/s42438-020-00184-7

**Published:** 2020-09-03

**Authors:** Mark Smith

**Affiliations:** Loughborough University, Loughborough, UK

**Keywords:** Covid-19, Digital, Education, Postdigital, Zine, Techlash

## The Pandemic Techlash

I was first made aware of Selwyn’s ability to ‘look beyond the usual teaching and learning concerns of “EdTech” research’ (Selwyn and Jandrić 2020) at a 2011 conference that was focused on the legacy of Marshall McLuhan. Selwyn’s work continues to stimulate critical debate in this area. Neil Selwyn has self-produced the *Techlash* zine as a global academic platform for critical commentaries on the state of EdTech—the concept that technology improves learning and teaching.

The first issue of *Techlash* investigates the state of EdTech during the Covid-19 pandemic. There is a sense of urgency within the zine that demands the reader’s scrutiny. It contains three articles: an introductory piece by Neil Selwyn lays the ground and fleshes out the zine’s challenge for a more a critical (dare one say, argumentative?) debate of the role of EdTech in a post-Covid world; Felicitas Macgilchrist presents informative narratives that include the social story of lockdown; and Ben Williamson shines the light on upon power networks and their attempts to control the roll-out of the new normal in education.

The concept of ‘Techlash’ comes from Adrian Wooldridge’s 2013 article for The Economist: ‘The coming tech-lash**:** The tech elite will join bankers and oilmen in public demonology’ (Woolridge 2013). Five years later, Rana Foroohar (2018) embroidered the term as: ‘the growing public animosity towards large Silicon Valley platform technology companies and their Chinese equivalents’. One immediately thinks of Google, Facebook, Microsoft, Huawei, and now TikTok. Proponents of open source alternatives and guardians of our privacy have argued vociferously against allowing Big Tech control of our data. We now see concern at government level, including the banning of Chinese business from national digital infrastructures, played out through the media.

Perhaps in the present Huawei-banned Silicon Valley, the term ‘Techlash’ now conjures up images of a rubber-clad TikTok-bashing Donald, whipping a Chinese actor dressed as Cogsworth. Even if you are not a Disney fan, you may remember the moustachioed clock in Disney’s *Beauty and the Beast* (Trousdale and Wise 1991)*.* Perhaps the clock cries out in pain, ‘Someone might break the spell any day now!’—a line from Human Again, a song left on the cutting floor before the film’s theatrical release (see Ghez 2010: 501). Perhaps the zine’s title is intended to hook in both critical EdTechers and those of us patiently waiting for a post-Trump Disney S&M reboot of *Beauty and the Beast*.

## Things Are *Not* Going to Be the Same

In the preface, Selwyn assumes that in a post-pandemic world ‘things are *not* going to be the same’ and that ‘important shifts in tone, pace and intent that need to be factored into any critical discussions of digital education’ (Selwyn, Macgilchrist, and Williamson 2020: 4). Selwyn further insists that the Covid-19 pandemic is ‘*not* used as an excuse to push through further corporate reforms of public education’ (6). Though Selwyn does not specifically mention late twentieth century commentators such as Jacques Rancière (1991) and his ‘ignorant schoolmaster’, or Sugata Mitra and his ‘hole-in-the-wall’ experiment (Mitra and Rana 2001), they are here in spirit.

The path to understanding that tech can (and must) replace in-person contact is both well-laid and tangible in the present circumstances. Whilst colleagues’ university contracts have not been renewed, significant budgets have undoubtedly been allotted to purchasing proprietary delivery options such as Microsoft Teams. In his article, ‘Digital education in the aftermath of Covid-19: critical concerns & hopes’, Selwyn puts meat on these bones. Simultaneously, he points to a hopeful future in which critically engaged publics are receptive ‘to difficult conversations about EdTech’ (9). I wholeheartedly agree with Selwyn in his call for scholars to counter the ‘re-imagining of public education’ as a ‘tech issue’ (10) in which off-the-peg digital systems will provide solutions for everyone and every situation. Education does need to be framed as a social concern. Without placing this understanding to the fore of critical education policy debate, we neglect our common history of progressive education. Dewey who?

## EdTech as ‘Translational’, ‘Relational’, and ‘Convivial’

Felicitas Macgilchrist continues with this critical and societal perspective on EdTech during the pandemic. She describes her ‘Three stories about EdTech after the Corona pandemic’ as ‘translational’, ‘relational’, and ‘convivial’. Translational EdTech is one for the instrumentalist technophiles—‘it’s cool to use tech’ (13). This echoes ‘classic progress narratives of the nineteenth century’—that ‘new tech is modern, it’s the future … and anything else is backward’ (13). Cue Marshall McLuhan, waving around his rearview mirror (McLuhan, Fiore, and Agel 1967: 100).

Macgilchrist’s ‘relational’ narrative focuses on ‘relationships, solidarity and creative communication’ (13). It is a story of ‘collaboration and social interaction while social distancing’ that we know through social media (14). She calls for a critical examination of those narratives that provide alternatives to the instrumentalism of EdTech gurus such as Sal Kahn. Macgilchrist’s third narrative—‘convivial EdTech’—is celebratory. It looks to the role of EdTech in ‘transforming social, cultural, ecological and economic hierarchies’ (14).

Macgilchrist provides the reader with an interesting and informative (if all too rapid) overview of the relationship between EdTech, society, and the critical scholar. It certainly had me looking up some of her references for further information. Her tangible narratives include moments of the micro-level, in which she asks us to consider lockdown from a personal perspective. For example, teachers who solve problems through using relationships with children and their parents, through telephone conversations and paper, rather than global corporate policy. Above all, she restates a sociological understanding that ‘apparently mundane practices are profoundly political’ (16). Regardless of our critical scrutiny of key actors within EdTech policy, the ‘personal’ remains ‘political’ (Hanisch 1969).

## Power Networks

The final article, Ben Williamson’s ‘New pandemic EdTech power networks’, investigates the positioning of major players, principally UNESCO, the World Bank, and the OECD. The piece is full of details that may be unfamiliar to many readers—there is little room for anecdote or polemic here. Williamson maps out the activities of these players during the pandemic, leading our understanding of how ‘emerging networks of organizations’ are seeking to solve the global disruption of education, whilst simultaneously paving the way ‘for longer-term transformations to education systems, institutions and practices’ (19).

Williamson speculates that the global education policy is preoccupied with delivering ‘schooling without schools and degrees without campuses’ (20). Digital technology and online learning have been perceived as the primary policy—the solution to lockdown measures. He indicates the level of policy shift that has occurred, ‘almost without contest, despite years of critical studies of international organisations such as OECD and World Bank’ (27). In contrast to the local action narrated by Macgilchrist, this is a scene in which these new power networks operate at a global level, ‘seeking to build a private infrastructure on which public education will depend’ (27). Considering the alacrity with which some university colleagues have touted on behalf of Microsoft Teams, these networks have deep roots, nurtured through decades of embedding proprietary systems within education.

## Conclusion

**Fig. 1:**
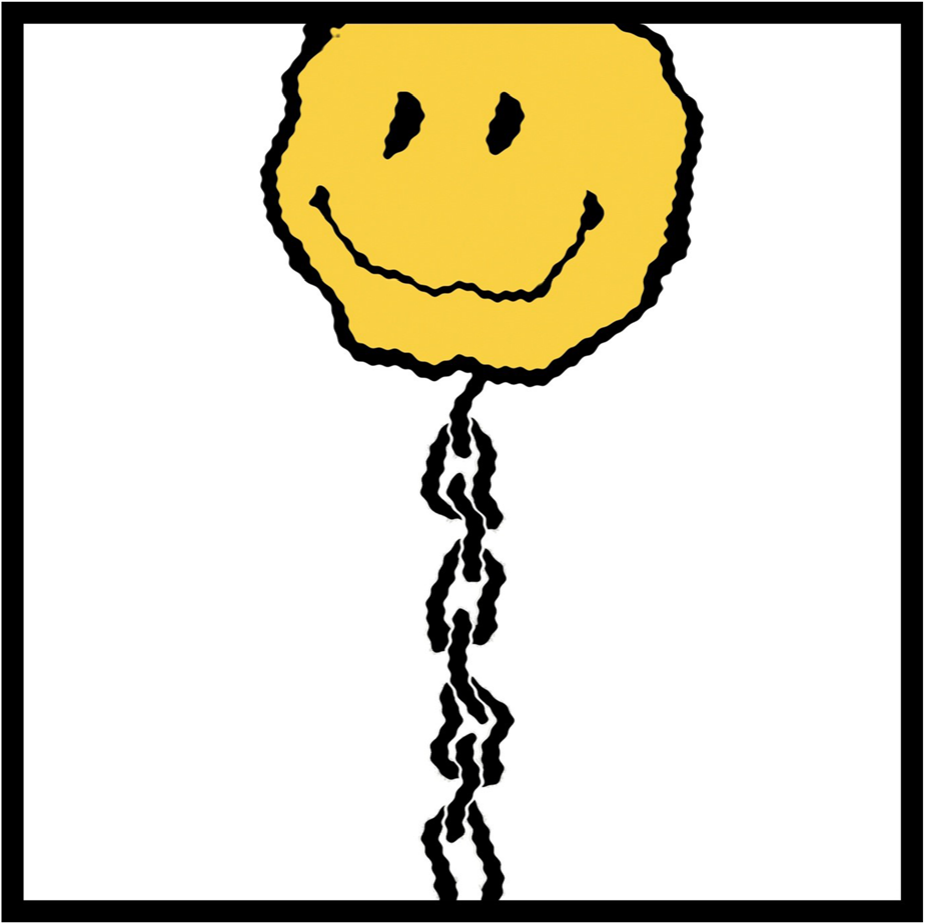
Free at Last! © Mark Smith 2020, reproduced with author’s permission

This is an interesting read, written by three very different critical scholars. I particularly like the short format as it makes for an easy comparison between the three sets of information. Also, *Techlash* is a free, DIY publication. In itself, that is to be lauded. I would suggest that Selwyn opens up the zine to artists, illustrators, and designers who want to contribute to the critical discussion of the relationship between technology and education. Read my contribution in this regard (see Fig. 1) as the deflation of our supposed optimism in tech to liberate us.

Regarding the content, were it not for my own recent experiences during lockdown, I might be more sceptical. For example, the focus of university management on ‘how’ to use Teams, as opposed to ‘why’—or even, ‘why *Teams*?’. Selwyn, Macgilchrist, and Williamson (2020) present different aspects of a critical debate in which we are all involved. There is a sense of urgency in *Techlash* that demands our response. Above all, I would recommend readers to use Neil Selwyn’s new zine format as a platform for future discussion—for critical collaboration. This is why he has produced *Techlash*.
